# Overexpression of Pregnancy Zone Protein in Fat Antagonizes Diet-Induced Obesity Under an Intermittent Fasting Regime

**DOI:** 10.3389/fphys.2022.950619

**Published:** 2022-08-16

**Authors:** Xiaoxiao Jiang, Jun Lin, Meng Dong, Xiaomeng Liu, Yuanyuan Huang, Hanlin Zhang, Rongcai Ye, Huiqiao Zhou, Chunlong Yan, Shouli Yuan, Li Chen, Rui Jiang, Kexin Zheng, Wanzhu Jin

**Affiliations:** ^1^ Key Laboratory of Animal Ecology and Conservation Biology, Institute of Zoology, Chinese Academy of Sciences, Beijing, China; ^2^ Guangdong Provincial People’s Hospital, Guangdong Academy of Medical Sciences, Guangzhou, China; ^3^ University of Chinese Academy of Sciences, Beijing, China; ^4^ Institute of Neuroscience and Translational Medicine, College of Life Science and Agronomy, Zhoukou Normal University, Zhoukou, China; ^5^ Agriculture College of Yanbian University, Yanji, China; ^6^ Institute of Infectious Disease, Ditan Hospital, Capital Medical University, Beijing, China

**Keywords:** intermittent fasting regimen, pregnancy zone protein, white adipose tissue, beige, vascularization

## Abstract

The intermittent fasting regimen (IFR) has been certified as an effective strategy for improving metabolism. But the underlying mechanism is still obscure. Beige induction in white adipose tissue (WAT) by IFR may account for this. It has been demonstrated that the erupting of pregnancy zone protein (PZP) from the liver coincides with membrane translocation of grp78 in brown adipocytes during IFR to activate brown adipose tissue (BAT), which may partly explain the metabolic benefits of IFR. Liver-derived PZP appears to be responsible for all metabolic regulatory functions; the effect of boosting energy expenditure disappeared in liver-deficient mice. To verify whether any liver-specific modification was essential for functional PZP, we used the PZP adipose tissue-specific overexpression mice model (PZP TG). We found that the metabolic disorders induced by high-fat diet were improved in PZP TG mice under IFR. Additionally, in addition to the activation of BAT, UCP1 protein and angiogenesis were increased in WAT, as well as the expression of genes associated with glucose utilization. These results demonstrate that PZP fat-specific TG increased the energy conversion of WAT, indicating that WAT may be another direct target for PZP during IFR.

## Introduction

The increasing consumption of high-energy fast foods and the prevalence of sedentary lifestyles are accelerating the global epidemic of obesity; it has replaced under-nutrition and infectious diseases as one of the most significant contributors to poor health (Chavasit et al., 2013). Adipose tissue expansion is the main characteristic of obesity. Evidence shows that adipose tissue is not only simply an energy store but also an essential functional organ in the regulation of systemic homeostasis by independently responding to outside stimuli and secreting factors in collaboration with other tissues (Sakers et al., 2022). Inflammation and hormone insensitivity of adipose tissue is often accompanied by metabolic disorders (Trayhurn, 2013). Positive and negative regulatory properties of adipose thermogenesis play pivotal roles in whole-body metabolic homeostasis (Huang et al., 2022). Therefore, adipose tissue may be an important target for intervention in metabolism-related diseases.

The intermittent fasting regimen (IFR) is characterized by a longer fasting window, and its effect on extending longevity has been proven (Longo and Mattson, 2014; Ulgherait et al., 2021). There is now a consensus that IFR can improve the metabolic disorders of obese people. Increasing vascular endothelial growth factor (VEGF) secretion by adipocytes during IFR may account for this (Kim et al., 2017). VEGF-induced angiogenesis in adipose tissue increases thermogenesis and decreases tissue inflammation, thus counteracting obesity and insulin resistance induced by diet (Cao, 2013; Sung et al., 2013). Blocking the VEGF-VEGFR2 pathway *via* antibody or antagonist suppressed white adipose tissue (WAT) browning/beiging (Robciuc et al., 2016). Acetate and lactate produced by gut microbiota under IFR may also contribute to WAT browning (Li et al., 2017b). CDC-like kinase 2 induced by refeeding in brown adipose tissue (BAT) may limit diet-induced thermogenesis (Hatting et al., 2017). Before that, heat production of BAT was increased under refeeding stimulation (Lin et al., 2021). The underlying mechanism by which IFR provides metabolic regulatory benefits remains obscure, but IFR shows promise for suppressing obesity.

Pregnancy zone protein (PZP), a hepatokine promoted by refeeding, has been demonstrated to play a key role in diet-induced thermogenesis *via* increasing UCP1 protein in BAT. PZP gets its name because it increases 100 times during pregnancy. Studies have shown that PZP acts as a molecular chaperone in late pregnancy to protect the fetus from the maternal immune system, showing its plasticity in anti-inflammatory activity, suggesting other pathological conditions of immune disorders, such as aging and obesity, might be related to the negligence of PZP protein. Recently, a GWAS analysis found a correlation between the level of PZP and aspartate aminotransferase (AST), suggesting that it might be an invasive biomarker in predicting liver fibrosis. In addition, in a large multi-center diet intervention study (DiOGenes), the authors analyzed the blood proteins of the volunteers who lost weight on a low-calorie diet and were given low carbohydrate and high protein to maintain weight loss for 12 months, and the level of PZP protein in the successful maintenance group was significantly higher than that in the weight loss group. These findings lead us to speculate that the metabolic regulation function of PZP protein may not be limited to BAT tissues.

A previous study reported that administration of PZP helps protect mice against adiposity and glucose intolerance induced by high-fat diet (HFD), accompanied by improved inflammation and decreased weight of white adipose tissue, indicating that WAT may also be the target of PZP during IFR. To verify this hypothesis, we constructed a PZP adipose tissue overexpression transgenic mouse model (PZP TG) and found that mice that specifically expressed PZP in adipose tissue were more resistant to HFD-induced obesity.

To our surprise, the expression of beige-related genes and the glucose metabolism-related genes were significantly increased in PZP TG mice under IFR, accompanied by the increasing vascularization of WAT in PZP TG mice. These results demonstrate that overexpression of PZP in adipose tissue could improve energy metabolism *in situ*.

## Materials and Methods

### Mice

C57BL/6J mice were purchased from Vital River Laboratory Animal Technology Co., Ltd. To generate adipose tissue-specific PZP transgenic (PZP TG) mice, the DNA fragment of the adipose-specific promoter/enhancer from the adiponectin gene together with the PZP-coding sequence was guided into C57BL/6J mice *via* a shotgun method at the Institute of Zoology, Chinese Academy of Sciences. PZP TG-positive mice were mated with wild-type (WT) mice to generate PZP TG and littermate WT control mice. The mice were housed in an SPF laboratory animal-house (Institute of Zoology of Beijing, Chinese Academy of Sciences, China) at room temperature (24°C) with a 12-h light/dark cycle. The high-fat diet (HFD) comprises 60% fat, 20% kcal from carbohydrates, and 20% from protein (MD12033, Medicience). All animal studies were approved by the Institutional Animal Care and Use Committee of the Institute of Zoology, Chinese Academy of Sciences.

### Intermittent Fasting Regimen

We used a 1:3 diet regimen as in our previous study. In a whole IFR cycle, the mice were food deprived for 1 day and then re-fed for 3 days. This regimen provided the mice with sufficient time to compensate for the reduced amount of food intake after 1-day fasting. No difference in cumulative food intake between the IFR group and normal diet group enabled us to examine the effects of IFR, independent of caloric intake difference.

### Body Composition Measurements

The total fat and lean mass of mice were measured by the Small Animal Body Composition Analysis and Imaging System (MesoQMR 23 060H-I; Nuimag Corp., Shanghai, China), according to the manufacturer’s instructions (Lin et al., 2021).

### Metabolic Rate and Physical Activity

Oxygen consumption was measured when mice were fed upon HFD for 2 weeks without a difference in body weights. Mice were acclimated to the system, maintained at 24°C in a 12-h light/dark cycle for 20–24 h, and fasted VO_2_ and VCO_2_ of each mouse were measured during the next 24 h with food deprivation. Then, re-fed VO_2_ and VCO_2_ were obtained with free access to food and water. Physical activity was determined at 12 weeks of age with a TSE LabMaster (TSE Systems, Bad Homburg, Germany) (Lin et al., 2021).

### Glucose Tolerance Tests

After the last IF cycle, mice were subjected to a glucose tolerance test (GTT). The GTT was performed as follows: after food deprivation for 16 h, mice were intraperitoneally injected with glucose solution (1.5 g/kg body weight). Plasma glucose was measured with a glucose meter (Roche Diagnostics Corp) before and 15, 30, 60, 90, and 120 min after injection (Lin et al., 2021).

### Insulin Tolerance Tests

After the last IF cycle, mice were subjected to insulin tolerance tests (ITTs). ITTs were performed as follows: after food deprivation for 4 h, mice were intraperitoneally injected with 0.75 IU/kg. Plasma glucose was measured using a glucose meter (Roche Diagnostics Corp) before and 15, 30, 45, and 60 min after injection.

### Histological Analysis

For H&E staining, tissues fixed with 4% paraformaldehyde were embedded in paraffin. Sections (4-μm-thick) were stained and observed under a ×10 or ×20 objective lens. For tissue staining, WAT was dissected from EP and incubated on ice with 2% PFA overnight, followed by permeable treatment with 0.3% Triton X-100 and 3% FBS in PBS for 1 h at RT. Next, tissues were removed into a staining buffer containing the primary antibody (anti-CD31, ab182981) at 4°C for 24 h and then incubated with a fluorophore-conjugated secondary antibody. The whole EP was scanned under a confocal laser-scanning microscope (Zeiss LSM 780).

### Western Blotting

To detect relative protein expression levels, a series of Western blots were performed. Briefly, tissue proteins were obtained by additional chopping with a T10 basic ULTRA-TURRAX handheld homogenizer (IKA, Germany). Equal amounts of proteins were separated on 10% SDS–polyacrylamide gels and then transferred to PVDF membranes. The membranes with proteins were incubated with the following antibodies overnight at 4°C: anti-UCP1 (ab155117, Abcam), anti-VEGF receptor 2 (ab221679, Abcam), and anti-tubulin (2146, CST) and then were incubated with HRP-conjugated secondary antibodies for 1 h at room temperature. All signals were visualized and analyzed by densitometric scanning (Image Quant TL 7.0; GE Healthcare Biosciences, Uppsala, Sweden). The intensity values of the bands were analyzed using ImageJ software (National Institutes of Health, Bethesda, MD, United States).

### Real-Time qPCR

Total RNA from tissues and cells was isolated with TRIzol Reagent (Thermo Fisher Scientific, United States) and reverse-transcribed with high-capacity cDNA reverse transcription kit (Promega, United States). The relative expression of genes was detected by the real-time fluorescence quantitative polymerase chain reaction (qPCR) (Light Cycler 480, Roche, Sweden) with SYBR Green Master Mix (Promega, United States).

### Statistical Analysis

To each group, 7–11 mice were randomly assigned to reduce the individual differences. For the *in vitro* experiment, a minimum of four replicates were designed. Statistical analyses were performed with GraphPad Prism version 9.0. Outliers were evaluated using the ROUT method (Q = 1%). Data were expressed as mean ± SEM. Unpaired two-tailed Student’s *t*-test was used for two-group comparison. *p*-values were calculated to determine statistical differences. ***p < 0.001, **p < 0.01, and *p < 0.05 were considered significant.

## Results

### The Anti-Obesity Effect of an Intermittent Fasting Regimen Was Enhanced in Pregnancy Zone Protein Transgenic Mice

PZP is mainly expressed in the liver and a small amount in the kidney, immune cells, and brain tissue. Our previous studies have shown that liver-derived PZP undertakes almost all metabolic regulatory functions regulated by IFR. To investigate whether this difference in function is caused by tissue-specific modification, we constructed a transgenic mice model that specifically overexpresses PZP in adipose tissue under the regulation of adiponectin promoter (PZP TG mice).

**FIGURE 1:**
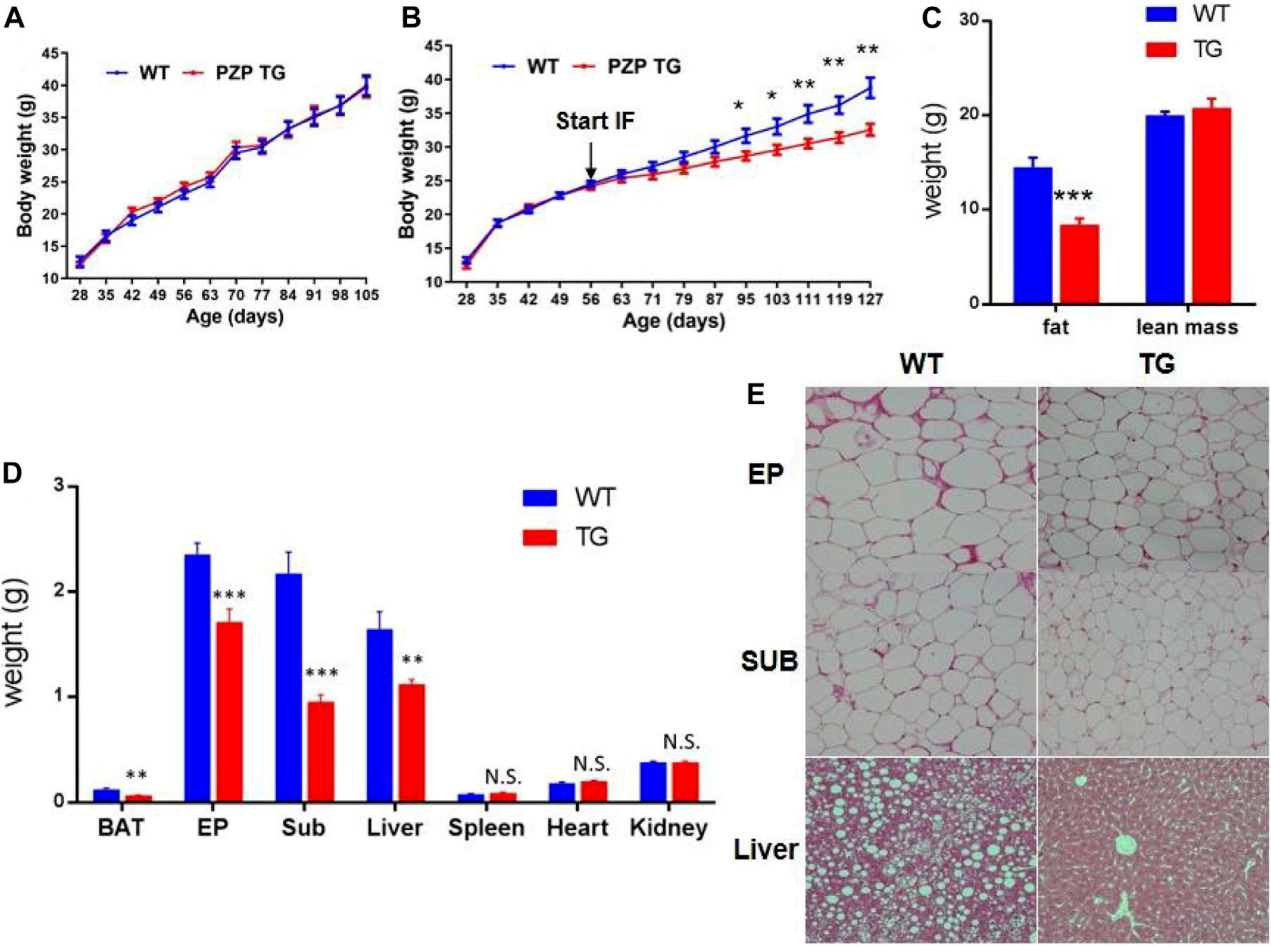
Anti-obesity effect of IFR is enhanced in PZP TG mice. **(A)** Body weight of WT and PZP TG mice under HFD (*n* = 10). **(B)** Body weight of WT and PZP TG mice under HFD and IF (*n* = 12). **(C)** Body composition of WT and PZP TG mice when mice were fed on HFD and IF for 12 weeks (*n* = 11). **(D)** Tissue weight of WT and PZP TG mice (*n* = 8). **(E)** Representative hematoxylin–eosin (HE) staining images of iWAT, eWAT, and liver from WT and PZP TG mice showing that PZP TG mice under IF conditions had smaller adipocytes and less lipid accumulation in the liver (scale bar = 100 μm).

As the following results show, overexpression of PZP in adipose tissue did not affect body weight gain of mice fed with a high-fat diet containing 60% fat (Figure 1A). For the same period induced by the high-fat diet, mice on the intermittent fasting diet gained less weight than those on the normal diet, and PZP TG in adipose tissue amplifies the anti-obesity effects of the intermittent fasting diet (Figure 1B), and such weight change was mainly caused by the reduction of fat proportion but did not affect the lean mass of mice (Figure 1C). These results indicated that PZP TG mice were less sensitive to HFD-induced obesity.

According to our further measurements, the weight of BAT, SUB, EP, and liver was significantly lighter in TG mice than in WT mice (Figure 1D). The results of tissue section experiments directly demonstrated that PZP TG mice had less fat storage in fat and liver cells under the dietary measure (Figure 1E). Taken together, the overexpression of PZP in adipose tissue helps anti-diet-induced obesity (DIO), and there are no necessary modifications of PZP by the liver for metabolic regulation.

### Pregnancy Zone Protein-Meditated Improvements in White Adipose Tissue Metabolic Activity Depend on Energy Expenditure

**FIGURE 2:**
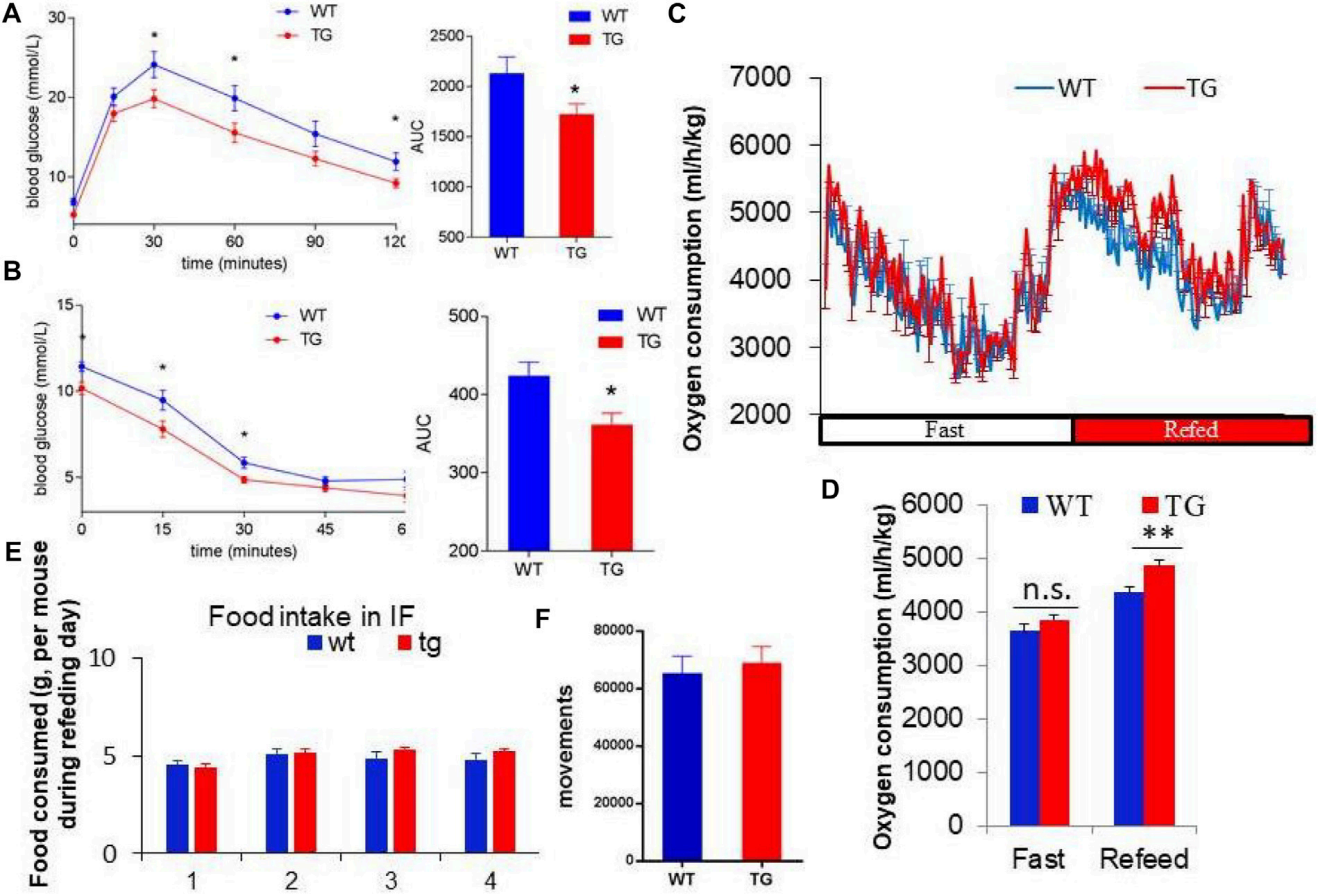
PZP-meditated improvements in metabolism depend on energy expenditure. **(A,B)** Blood glucose concentration at each time point and area under the curve of the glucose tolerance test (GTT) **(A)** and insulin tolerance test (ITT) **(B)**. **(C,D)** Oxygen consumption rate **(C)** and energy expenditure **(D)** of WT and PZP TG mice showed that PZP TG leads to a marked increase in whole-body energy expenditure (*n* = 7). **(E,F)** Food consumption **(E)** and physical activity **(F)** of WT and PZP TG mice (*n* = 9).

To explore the metabolic effects of overexpression of PZP in adipose tissue, GTT and ITT tests were used to measure the glucose utilization rate of mice after 10 weeks of IF treatment. PZP TG mice showed increased ability to clear exogenous glucose (Figure 2A) and were more sensitive to insulin stimulation (Figure 2B). These results indicate that overexpression of PZP in adipose tissue improves the metabolic disorder induced by HFD.

Furthermore, we found that the overall energy metabolism of TG mice was increased (Figures 2C,D), while the food intake and exercise of PZP TG mice were not changed compared with WT mice (Figures 2E,F). These results suggest that the anti-obesity and metabolic improvement effects caused by PZP TG depend on the increase in diet-induced energy expenditure.

### Brown Adipose Tissue Activity was Increased in Pregnancy Zone Protein Transgenic Mice

**FIGURE 3:**
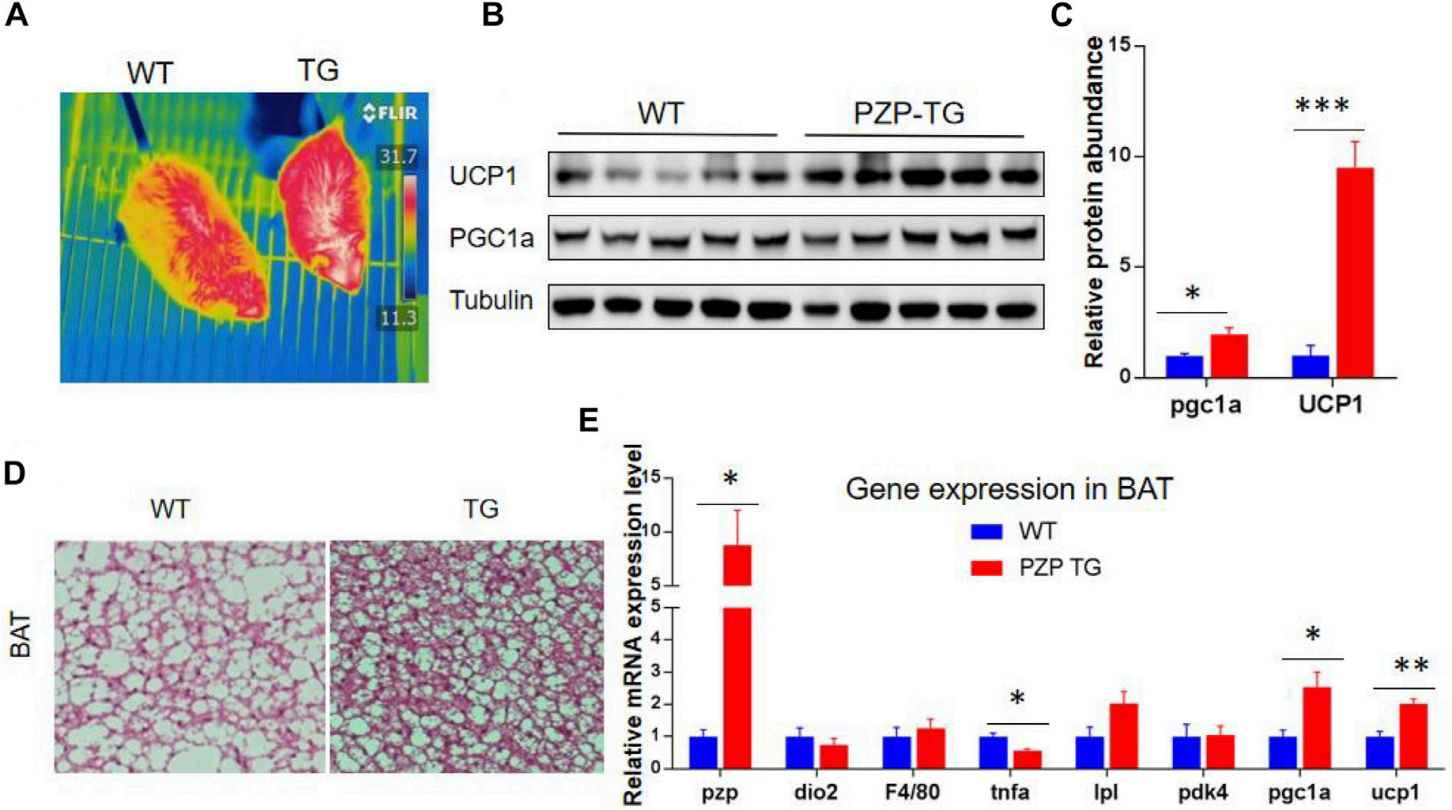
BAT activity was increased in PZP TG mice. **(A)** Surface temperature of WT and PZP TG mice after refeeding time was measured by the infrared imager. **(B,C)** Immunoblots of total BAT lysates from WT and PZP TG mice showed UCP1 and PGC1a levels increased in PZP TG mice (*n* = 5). **(D)** Representative H&E staining images of BAT from WT and PZP TG mice show that PZP TG mice under IF conditions had smaller adipocytes and less lipid accumulation in the liver (scale bar = 100 μm). **(E)** Relative mRNA expression of adipogenic, thermogenic, and inflammatory genes in BAT from WT and PZP TG mice (*n* = 7).

BAT is the major organ of adaptive thermogenesis, and thermogenesis was inhibited under IF when PZP was deleted (Lin et al., 2021). To explore the effects of adipose tissue-derived PZP on BAT, PZP TG mice and WT mice were subjected to HFD and IF stimulation. Under the monitoring of an infrared camera, we found that the temperature of the interscapular region of PZP TG mice was significantly higher than that of wild-type mice (Figure 3A), suggesting more heat production in BAT of TG mice.

BAT heat generation mainly depends on UCP1 protein to convert the chemical energy generated by the hydrolysis of fat and carbohydrate into heat. Subsequent molecular experiments demonstrated that the transcription of heat production-related genes and the abundance of proteins, such as UCP1 and PGC1a, in BAT of PZP TG mice were significantly increased (Figures 3B,C,E). Tissue section experiments showed that fat accumulation in BAT cells was significantly reduced (Figure 3D). These results indicate that PZP TG significantly increased the thermogenic activity of BAT.

### Pregnancy Zone Protein Transgenic Promotes SUB Remodeling Against High-Fat Diet

**FIGURE 4:**
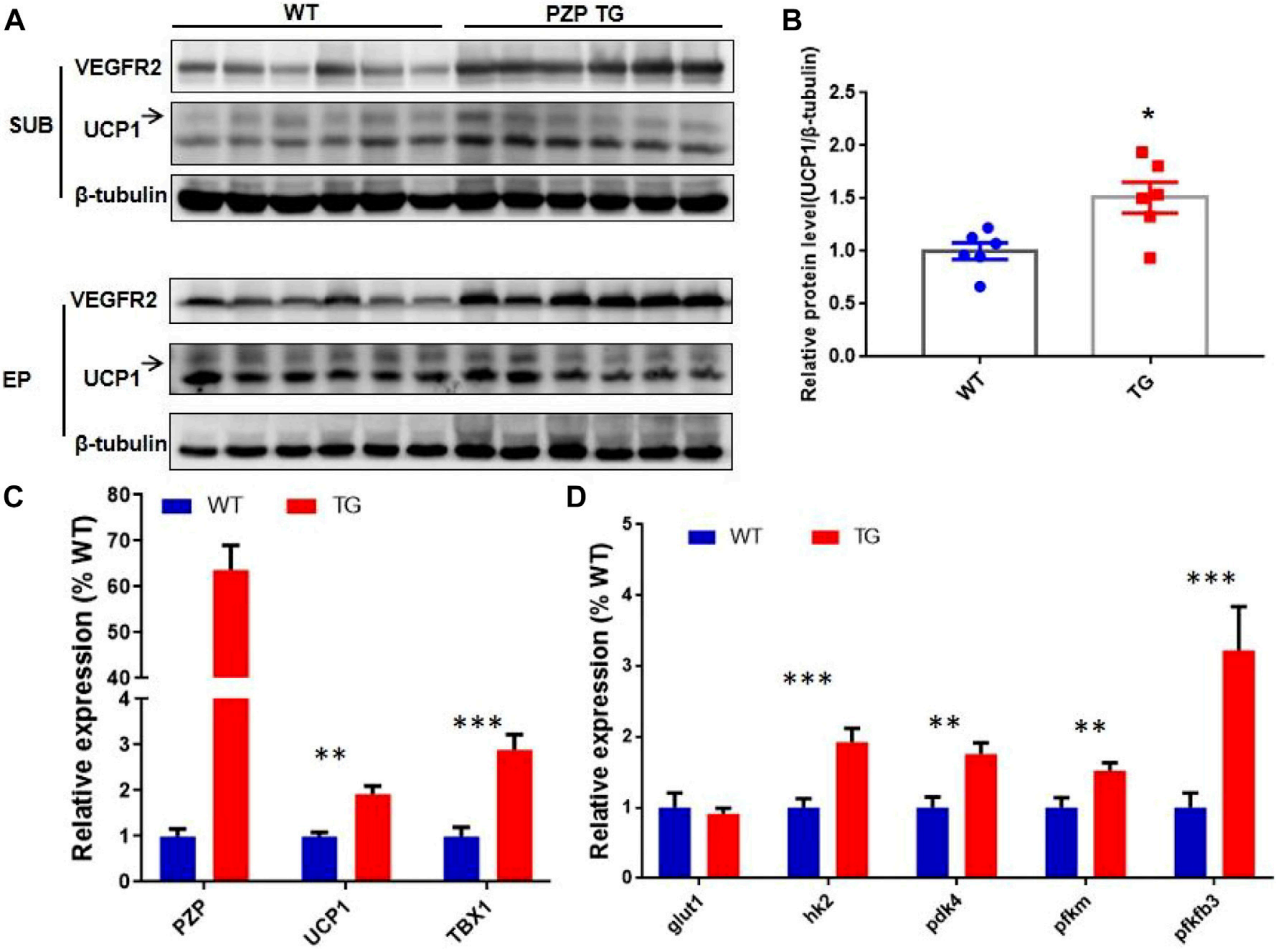
PZP TG promotes WAT remodeling against HFD. **(A)** Immunoblots of total iWAT and vWAT lysates from WT and PZP TG mice using indicated antibodies. **(B)** UCP1 protein abundance of iWAT from TG mice was significantly higher than that from the WT mice. **(C)** Relative mRNA expression of the mentioned genes in SUB from WT and PZP TG mice (*n* = 7). **(D)** Relative mRNA expression of glucose metabolism-related genes in iWAT from WT and PZP TG mice (*n* = 7).

In addition to the classic BAT, the beige cells present in white adipose tissue can also specifically express UCP1 protein, thus promoting energy consumption and heat release. The discovery of beige fat in human has made it a hot topic in treating obesity. To explore whether PZP can induce thermogenesis in white adipose tissue, we detected UCP1 protein in subcutaneous and epididymis white adipose tissue. Under the condition of IF treatment, a small amount of UCP1 protein was expressed in mice, while the abundance of UCP1 protein in SUB of PZP TG mice was significantly higher than that of WT mice (Figures 4A,B). Meanwhile, in SUB, the mRNA level of TBX1, the main gene for inducing beige production, was significantly increased, indicating the potential for PZP TG to induce beige production (Figure 4C). Vascular formation and UCP1 protein expression are the main factors promoting WAT thermogenesis during IFR. Our results show that the abundance of VR2 (VEGF receptor 2), a signature protein for angiogenesis, was significantly increased in PZP TG mice (Figure 4A). Furthermore, we found that the expression of key enzymes involved in glycolysis was significantly upregulated in TG mice (Figure 4D), suggesting that PZP TG accelerated glucose metabolism in mice. In conclusion, overexpression of PZP in adipose tissue could increase energy dissipation by accelerating glucose metabolism and heat production in white adipose tissue.

### Pregnancy Zone Protein Transgenic Promotes Angiopoiesis in EP

**FIGURE 5:**
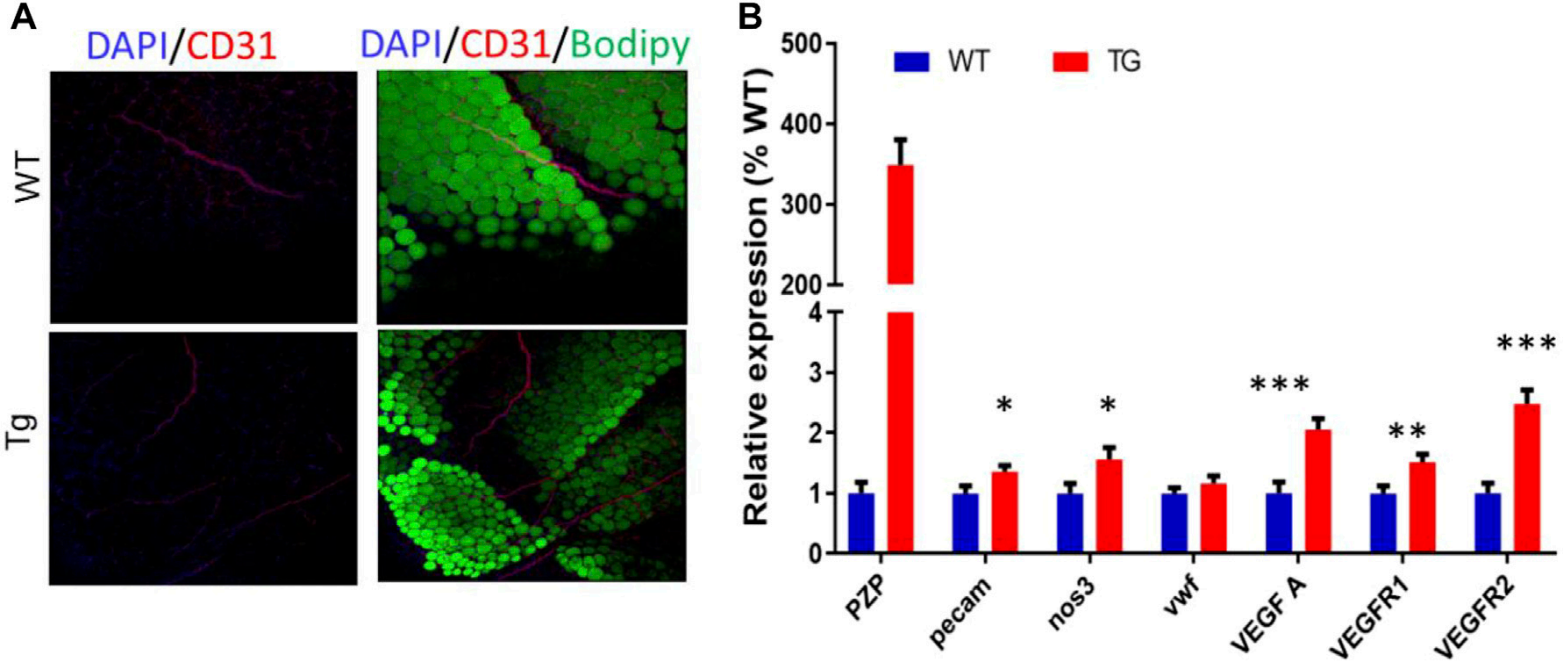
PZP TG promotes angiopoiesis in epididymis adipose tissue. **(A)** Whole-mount tissue staining images of vWAT from WT and PZP TG mice using anti-CD31. **(B)** Relative mRNA expression of angiopoiesis-related genes in EP from WT and PZP TG mice (*n* = 7).

As in subcutaneous adipose tissue, the VR2 protein was increased in epididymis adipose tissue between the two groups. To further verify the effect of PZP TG on vascular formation in mice, whole-tissue staining was performed on flat areas of EP from TG and WT mice (Figure 5A). Confocal microscopy showed that the number of blood vessels in EP of TG mice was significantly higher than that of WT mice. Subsequently, the qPCR test showed that VEGFA and PECAM, which promote angiogenesis, were significantly upregulated in the EP of TG mice (Figure 5B). These results demonstrate that PZP in adipose tissue could promote vascular formation in WAT.

## Discussion

It has been demonstrated that liver-derived PZP amplified the weight loss effect of IFR (Lin et al., 2021), but it is unclear whether the metabolic regulation of PZP requires liver tissue-specific modification. This study found that the overexpression of PZP in adipose tissue can also activate the thermogenesis effect of BAT. More interestingly, the beige fat formation in the subcutaneous adipose tissue and vascularization in the subcutaneous and epididymal visceral adipose tissue was increased in PZP TG mice, demonstrating PZP itself regulates energy conversion of adipose tissue types under IFR.

Vasculation of adipose is often a co-occurring phenomenon with browning induced by external cues, which is dependent on the VEGF-VEGFR2 signaling but not UCP1 (Seki et al., 2016). In this study, we found that the expression of VEGFR2 in WAT was markedly upregulated in PZP TG mice under IFR. This demonstrates that the PZP-induced thermogenesis was not only dependent on UCP1 but also on angiogenesis in adipose tissue. Our mice model phenotypically resembles the VEGFA adipose-specific transgenic mice in which local upregulation of VEGFA in adipocytes improves vascularization and causes a “browning” of WAT, accompanied with upregulated UCP1 and PGC1α (Sun et al., 2012). Interestingly, Sun et al. (2014) continued to study depot effect using the BAT-specific VEGFA expression mice model and found that locally overexpressed VEGFA in BAT was easier to clarify the role of BAT activation than the previous model. Although PZP and VEGFA are both secreted proteins, expression at different locations could still reflect tissue specificity.

Meanwhile, recent studies have reported that glycogen metabolism in WAT was required for UCP1 expression that is depended on the P38 MAPK pathway (Keinan et al., 2021). Our study found that glucose metabolism was more active in PZP TG mice, suggesting that PZP may be an important link between glucose metabolism and beige formation. As an immunosuppressant, PZP protects the fetus from the mother’s immune system; our results confirmed that PZP overexpression significantly alleviated high-fat-diet-induced inflammation in WAT. These findings also enlighten us that other immune disorders, such as aging and diabetes, may be related to impaired PZP function.

Recently, PZP has been reported to act as a potential biomarker for screening the risk of lung adenocarcinoma (Yang et al., 2021) or prognosis of hepatocellular carcinoma (Zhang et al., 2019) in patients with type 2 diabetes. Subsequent studies have reported that the expression of PZP in tumor tissues was lower than that in normal tissues, and the loss of PZP would promote the progression of breast cancer (Zheng et al., 2018; Wu et al., 2021), laying the foundation for the potential of PZP protein in tumor therapy. These emerging studies show that PZP is important for the maintenance of normal body functions and deserves further study. To rule out the effects of food intake, we used a 1:3 diet regimen. A prolonged high-fat diet may attenuate IF cycle-induced thermogenesis (Wang et al., 2013), which is probably one of the reasons for not detecting beige fat in epididymal visceral adipose tissue. IFR with a shorter refeeding duration, such as fasting every other day (Li et al., 2017a) and every 2 days, might be more effective at inducing PZP-mediated mobilization of metabolic activity.

Many trials proved that IFR could effectively reduce body weight and improve cardiovascular events, diabetes, and other symptoms (Patterson and Sears, 2017; Harney et al., 2021; Maifeld et al., 2021). Several prospective clinical studies have counted different IFR effects and claim the superiority of one regimen or another for metabolic disorders. But, the lack of randomized control and the various eating time windows among studies make horizontal comparisons between studies difficult; selecting which regimen is the best remains unclear.

In recent years, several clinical studies with strict eating windows have quantified the effects of IFR. Sutton et al. (2018) reported that, compared with a 12-h eating window, eating for 6 h per day (8:00 a.m.–3:00 p.m.) could significantly improve the insulin sensitivity of prediabetic patients, islet *β*-cell insulin secretion, oxidative stress, and blood pressure.

A single-arm study by Wilkinson et al. (2020) demonstrated that a 10-h eating window and 14-h fasting regimen for 12 weeks resulted in a 3% average weight loss, a 4% reduction in waist circumference, and improvements in blood pressure and cholesterol in patients with metabolic syndrome. Duration of eating, rhythm cycle, and food composition significantly affected IFR (Ulgherait et al., 2021); large-scale randomized controlled trials involving volunteers and long-term follow-up carried out in multi-center cooperation will provide objective advice for establishing effective dietary strategies.

In summary, we report that local expression of PZP in WAT significantly induces beige browning as well as angiogenesis in adipose. Unlike previous studies, in which liver-derived PZP appeared to promote only BAT thermogenesis, our results suggest that WAT browning also plays an important role in the promotion of energy metabolism by PZP.

## Data Availability

The original contributions presented in the study are included in the article/Supplementary Material; further inquiries can be directed to the corresponding author.
